# No difference in frontal cortical activity during an executive functioning task after acute doses of aripiprazole and haloperidol

**DOI:** 10.3389/fnhum.2015.00296

**Published:** 2015-05-26

**Authors:** Ingeborg Bolstad, Ole A. Andreassen, Inge R. Groote, Beathe Haatveit, Andres Server, Jimmy Jensen

**Affiliations:** ^1^NORMENT, KG Jebsen Centre for Psychosis Research, Division of Mental Health and Addiction, Oslo University HospitalOslo, Norway; ^2^Institute of Clinical Medicine, University of OsloOslo, Norway; ^3^Department of Psychology, Institute of Social Sciences, University of OsloOslo, Norway; ^4^Department of Neuroradiology, Oslo University HospitalOslo, Norway; ^5^Centre for Psychology, Kristianstad UniversityKristianstad, Sweden

**Keywords:** dopamine, aripiprazole, haloperidol, executive function, healthy volunteers, fMRI

## Abstract

**Background**: Aripiprazole is an atypical antipsychotic drug that is characterized by partial dopamine D2 receptor agonism. Its pharmacodynamic profile is proposed to be beneficial in the treatment of cognitive impairment, which is prevalent in psychotic disorders. This study compared brain activation characteristics produced by aripiprazole with that of haloperidol, a typical D2 receptor antagonist, during a task targeting executive functioning.

**Methods**: Healthy participants received an acute oral dose of haloperidol, aripiprazole or placebo before performing an executive functioning task while blood-oxygen-level-dependent (BOLD) functional magnetic resonance imaging (fMRI) was carried out.

**Results**: There was a tendency towards reduced performance in the aripiprazole group compared to the two other groups. The image analysis yielded a strong task-related BOLD-fMRI response within each group. An uncorrected between-group analysis showed that aripiprazole challenge resulted in stronger activation in the frontal and temporal gyri and the putamen compared with haloperidol challenge, but after correcting for multiple testing there was no significant group difference.

**Conclusion**: No significant group differences between aripiprazole and haloperidol in frontal cortical activation were obtained when corrected for multiple comparisons. This study is registered in ClinicalTrials.gov (identifier: 2009-016222-14).^1^

## Introduction

Cognitive impairment is recognized as an important characteristic of psychosis, being present in schizophrenia patients and in high-risk individuals(Schaefer et al., 2013; Bora et al., 2014; Fatouros-Bergman et al., 2014). Cognitive deficits are good predictors of functional outcome in schizophrenia, and therefore an important treatment target (Green, 1996). There has been great effort to determine the effects of different types of antipsychotics on cognitive dysfunction, which together with negative symptoms, is far more difficult to treat than positive symptoms. The effect of antipsychotics on cognitive impairment is weak to moderate (Cuesta et al., 2001; Davidson et al., 2009). However, there is a large variation in the results on important cognitive domains such as executive function, attention and memory (Désaméricq et al., 2014); this may reflect different pharmacodynamic profiles. In general, it is suggested that atypical antipsychotics with relatively low dopamine and high serotonin receptor affinity improve cognitive functioning compared to typical antipsychotics with strong dopamine antagonism (Mishara and Goldberg, 2004; Désaméricq et al., 2014), and this has been associated with increased frontal cortical activation (Liemburg et al., 2012).

The antipsychotic effect of antipsychotic drugs is suggested to be directly related to dopamine D2 receptor affinity (Seeman and Lee, 1975). The Dopamine hypothesis of schizophrenia postulates that psychosis is due to dopamine system disturbances influenced by multiple risk factors including genetic and social environmental factors, in interaction with other neurotransmitter system disturbances (Howes and Kapur, 2009). The disturbances manifest as hyperactivity in the striatum, mainly associated with increased presynaptic dopamine synthesis capacity (Howes et al., 2012), and hypoactivity in the frontal cortex that is thought to be related to the striatal dopaminergic dysfunction (Meyer-Lindenberg et al., 2002); the latter issue being somewhat inconsistent (Manoach, 2003).

Typical (first generation) antipsychotic drugs exhibit mainly high affinity dopamine D2 receptor antagonism, whereas atypical (second generation) antipsychotic drugs target several other receptor types in addition. Aripiprazole is a relatively new drug that by some patients is tolerated better than other atypical antipsychotics because it gives less metabolic side effects (weight gain, dyslipidemia and diabetes mellitus; Citrome et al., 2014). In addition to having a relatively strong affinity for serotonin (5-hydroxytryptamine) 5-HT_2A_ and 5-HT_1A_ receptors, it is also a partial agonist at the dopamine D2 receptor. It is thought to display antagonistic properties in a dopamine-rich environment, and agonistic properties in a dopamine-deficient environment (Burris et al., 2002; Shapiro et al., 2003). Hence, aripiprazole has held promise for a more complete treatment of psychotic disorders, including schizophrenia (Tamminga, 2002). However, even though it effectively decreases positive symptoms, its efficacy in treating cognitive dysfunction and negative symptoms is less clear. The majority of studies report a variable degree of improvement (Schlagenhauf et al., 2010; Suzuki et al., 2011; Wang et al., 2013; Maat et al., 2014; Yeh et al., 2014), but cognitive impairment has also been found (Yasui-Furukori et al., 2012). To gain a better understanding of how antipsychotics affect cognitive functions it is crucial to disentangle the effects of illness and medication. One way to gain new insight is by studying effects of antipsychotics on cognition in healthy individuals.

Yet, only a few reports describe effects of antipsychotic drugs on cognition in healthy subjects. Decreased cognitive performance related to attention, response time and information processing have been reported for subjects given up to five doses of the typical antipsychotic drug haloperidol (Ramaekers et al., 1999; Saeedi et al., 2006; Vernaleken et al., 2006). Studies of atypical antipsychotics have shown impairments (Ramaekers et al., 1999; Morrens et al., 2007; amisulpride and olanzapine, respectively), no effect (Chung et al., 2012; aripiprazole) or improvement (Chung et al., 2012; amisulpride) on cognitive functioning. One functional magnetic resonance imaging (fMRI) study found no difference in neural activation when comparing sulpiride and placebo (Dodds et al., 2009). Aripiprazole have been examined in three neuroimaging studies this far. Using positron emission tomography (PET; Kim et al., 2013) found reduced frontal metabolism associated with extended response times. Another study showed increased striatal and decreased frontal regional cerebral blood flow (rCBF), presumably reflecting increased presynaptic dopamine synthesis and release in the striatum (Handley et al., 2013). Goozee et al. (2015) reported differences in neural activation between aripiprazole and haloperidol during a working memory task. More knowledge is needed about the effects of antipsychotic medication on cognition in general, and specifically about the functional brain activations related to antipsychotics with different pharmacodynamic profiles. The current study aimed to identify differences in brain activity related to cognition in healthy individuals given either aripiprazole or haloperidol. By using the Tower of London (ToL) task, targeting executive functioning (Sullivan et al., 2009), we directly compared brain activations associated by the two drugs in an fMRI experiment. We hypothesized that aripiprazole would yield a stronger task-related activation in the frontal cortex than haloperidol.

## Methods

### Participants and Medication

This study was approved by the Norwegian Regional Committees for Medical and Health Research Ethics, The Norwegian Medicines Agency and is registered in ClinicalTrials.gov (identifier: 2009-016222-14). All participants had responded to posted advertisement, gave written informed consent and were financially compensated. Participants were included if they passed somatic and psychiatric health screening, and had not taken psychotropic medication the previous 2 years or any drugs or medications the last 2 weeks. Three subjects were excluded based on abnormal electrocardiograms, and two based on structural brain abnormalities. The included subjects were randomized into one of three groups and given aripiprazole, haloperidol or placebo (eighteen individuals per group).

Subjects were challenged with one single dose at one time 4.8 (Range 4.0–5.6) hours prior to the fMRI scan. They were given either 10 or 15 mg of aripiprazole, 2 or 3 mg of haloperidol or placebo, all delivered as two or three pills based on their body weight (≤75 kg or >75 kg). Out of the first eight subjects that were given a drug, three subjects were unsuitable for scanning due to side effects. Therefore, the dose regime was subsequently lowered to 5 or 10 mg of aripiprazole or 1 or 2 mg of haloperidol.

Two datasets (one haloperidol, one aripiprazole) were excluded from analysis due to excessive head movement in the scanner (>3 mm), and one data set (aripiprazole) was lost due to technical problems. In total, 48 datasets were subjected to analysis. Table 1 displays demographic data and doses for subjects included in the analysis.

**Table 1:** **Demographical data and doses**.

	*n*	Age	Sex (males)	Doses (mg/kg)
Aripiprazole	14	25.9 (7.6)	7	0.11 (0.029)
Haloperidol	16	25.1 (7.1)	7	0.02 (0.007)
Placebo	18	23.5 (3.1)	8	

*Age and doses are given in means and standard deviations*.

### Experimental Task

**Figure 1:**
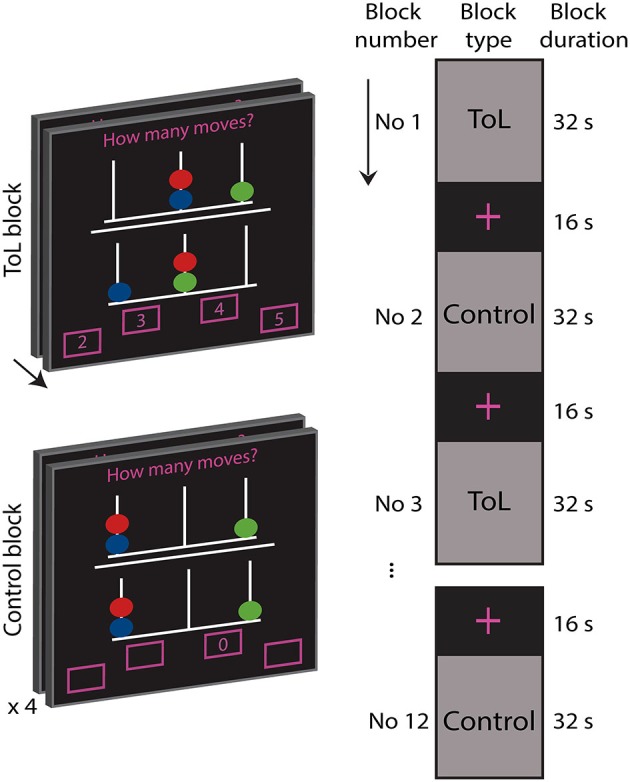
**Depiction of the experimental task**. During a task block the participants solved as many Tower of London problems as they could within 32 s. The object of the task was to mentally calculate how many moves (2, 3, 4, or 5) were needed to manipulate the balls on the lower pegboard to reach the goal configuration depicted in the upper pegboard, moving only one ball at a time. Answers were indicated by button presses of right and left thumbs and index fingers. Control blocks were comprised of four tasks of 8 s duration where subjects were asked to push the indicated button.

The ToL task is depicted in Figure 1 (Shallice, 1982). All participants completed a training version of the experimental task prior to the MRI scan. In the scanner, the subjects were first presented with an information screen, and asked to confirm that they understood the instructions before the task was presented.

### fMRI Data Acquisition

The task stimuli were presented through VisualSystem goggles and responses collected by ResponseGrip (Nordic Imaging Lab, Bergen, Norway). The E-Prime software (Psychology Software Tools Inc., Pittsburgh, Pennsylvania, USA) was used to present the task and to collect responses.

The MR examinations were performed on a 3T General Electric Signa HDxt scanner (GE Healthcare, Milwaukee, WI, USA). The blood-oxygen-level-dependent (BOLD) fMRI protocol consisted of a T2*-weighted echo-planar imaging (EPI) sequence in the transverse plane. The parameters were: repetition time (TR) = 2 s; echo time (TE) = 25 ms; flip angle (FA) = 78 degrees; matrix = 64; field-of-view (FOV) = 256 mm; slice thickness = 3.5 mm; gap = 0.5 mm; slices = 36. One run of 292 volumes was collected for each individual and the three initial volumes were discarded. Structural T1-weighted images were collected with the following parameters: TR = 7.7 s; TE = 3.0 s; FA = 12 degrees; matrix = 256; FOV = 256; slice thickness = 1.2 mm; slices = 172. These were obtained on a day prior to the fMRI and used in the functional image pre-processing and for radiological screening to identify subjects with structural brain abnormalities.

### Behavior Analysis

The behavioral data were analyzed using Statistical Package for the Social Sciences (SPSS, Version 21, SPSS Inc., Chicago, Illinois, USA). *T*-tests were used to analyze response times, while Wilcoxon signed rank tests and Mann-Whitney U tests were used to analyze accuracy and number of completed tasks. Spearman coefficients were used to describe associations between doses, beta values and behavioral data. Analyses of response times are based on problems with correct answers.

### Image Analysis

Data pre-processing and analyses was performed using the Statistical Parametric Mapping software 8 (SPM8)^2^ implemented in Matlab 7.5 (Mathworks, Natick, Massachusetts, US). Structural images were normalized to the Montreal Neurological Institute (MNI) reference brain (Evans et al., 1992). Functional images were aligned to the first volume of each time-series and spatially normalized using the parameters from the structural image normalization, resampled to 3 mm isotropic voxels, and smoothed using an 8 mm full-width at half-maximum Gaussian isotropic kernel and high-pass filtered using a 128 s cut-off value.

**Figure 2:**
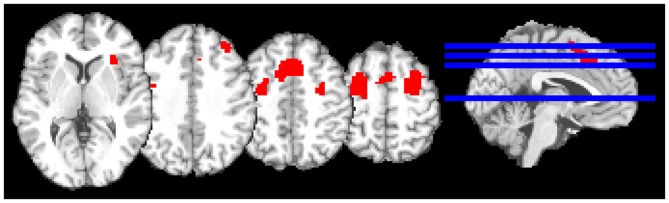
**The mask used in the region-of-interest (ROI) analysis**. The mask (displayed in red) was constructed of five activation clusters from the contrast of Tower of London task > Control task from the placebo group.

The model consisted of a canonical hemodynamic response function convolved with box-car functions for onsets for experiment and control conditions, in addition to movement parameters. Individual contrast images (ToL task > Control task) were moved to second-level random-effects analyses. To test for task effects *t*-tests (corrected for multiple comparisons at peak level threshold *p*_Family-wise error (FWE)_ < 0.05 and cluster size (*k*) > 20 voxels) were performed across all three groups and within each group. To test the hypothesis that haloperidol dampens activation more than aripiprazole in the frontal cortex during a ToL task, a voxel-wise small volume correction was used in a region-of-interest (ROI) analysis, followed by a test of whole-brain effects. Activations from the placebo group were used to construct a mask for the analysis of group effects between aripiprazole and haloperidol, ensuring that an area specifically targeted by this task served as ROI. In order to construct the mask, clusters in the frontal cortex obtained using correction for multiple comparisons for the whole brain were used. Five activation clusters were identified at peak level threshold *p*_FWE_ < 0.05, *k* > 20 for the contrast ToL task > control task, and combined into a binary mask constituting 971 voxels (Figure 2).

## Results

### Behavioral Findings

Behavioral data are given in Table 2.

**Table 2:** **Behavioral data for the tower of London task**.

	Accuracy (%)	Response times (ms)	Number of completed tasks				
			2 moves	3 moves	4 moves	5 moves	Total
Aripiprazole	77.8 (41.7–100.0)	7022 (1026)	6 (4–9)	6 (4–8)	6 (3–9)	4 (2–7)	22.5 (17–30)
Haloperidol	71.5 (52.4–100.0)	6217 (1295)	7 (5–10)	7 (3–10)	7 (3–10)	6 (2–8)	27.5 (16–36)
Placebo	80.1 (35.3–96.4)	6347 (1181)	7 (5–9)	8 (3–10)	7 (5–11)	6 (4–10)	26.0 (18–38)

*Accuracy and number of completed tasks are given in medians and range. Response times are given in means and standard deviations*.

Analysis of group effects revealed that the aripiprazole group completed fewer tasks than the haloperidol group (*U* = 64.50, *p* = 0.048). Comparisons between the drug and placebo groups were performed for completeness, and showed that the aripiprazole group completed fewer tasks than the placebo group (*U* = 67.00, *p* = 0.024). The numbers of completed tasks within each level of difficulty (2, 3, 4, or 5 moves) are given in Table 2.

There were no significant differences in accuracy or response times, and no behavioral effects between the haloperidol and placebo groups. In the placebo group there was a positive correlation between accuracy and average response time for correct answers (*ρ* = 0.64, *p* = 0.004), and a negative correlation between accuracy and number of completed tasks (*ρ* = 0.49, *p* = 0.040), while there were no correlations within the drug groups. To explore whether the drugs influenced performance, doses (mg/kg) of aripiprazole and haloperidol were correlated with the behavioral data. In the haloperidol group there was a negative correlation between accuracy and dose (*ρ* = 0.56, *p* = 0.025).

### fMRI Findings

**Figure 3:**
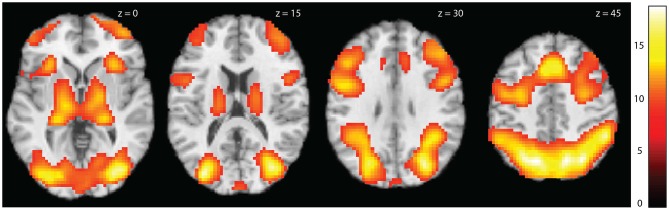
**Activation maps for task activations across all subjects**. Statistical parametric maps showing whole brain activations for the contrast Tower of London task > Control task across groups. Colors refer to *t*-values as coded in the bar to the right. The activations are shown at threshold *p*_FWE_ < 0.05, *k* > 20.

Whole brain analysis showed strong effects of task across all groups with prominent activations in the occipital, parietal and frontal cortex and in the thalamus (Figure 3). Within each group there were similar patterns, although with some variation (Table 3).

**Table 3:** **fMRI activation clusters**.

	Size	*p*	*Z*	*x*	*y*	*z*
**PLACEBO**
Middle frontal gyrus	380	<0.001	7.06	−30	5	58
Inferior occipital gyrus	2577	<0.001	6.71	36	−79	−8
Superior frontal gyrus	287	<0.001	6.16	30	−7	58
Cingulate gyrus	261	<0.001	6.16	−6	17	46
Midbrain	93	<0.001	5.94	−3	−22	−14
Cerebellum	373	<0.001	5.69	39	−64	−38
Thalamus	37	0.005	5.29	−24	−31	1
Insula	21	0.010	5.12	33	26	4
Cerebellum	80	0.011	5.10	−24	−37	−44
Middle frontal gyrus	22	0.014	5.03	39	38	40
**ARIPIPRAZOLE**
Precuneus	441	<0.001	6.71	18	−67	43
Precuneus	171	<0.001	5.95	−17	−70	43
Middle occipital gyrus	175	<0.001	5.76	−33	−82	13
Hippocampus	137	0.002	5.45	−24	−25	−5
Fusiform gyrus	30	0.002	5.41	48	−55	−20
Fusiform gyrus	40	0.003	5.37	−30	−61	−17
Medial frontal gyrus	39	0.008	5.18	0	20	49
Thalamus	43	0.010	5.15	15	−10	−2
**HALOPERIDOL**
Superior parietal lobule	954	<0.001	6.44	−27	−70	58
Angular gyrus	820	<0.001	5.96	30	−55	40
Superior frontal gyrus	78	<0.001	5.87	30	−4	58
Fusiform gyrus	59	<0.001	5.84	−30	53	−14
Middle frontal gyrus	43	<0.001	5.75	45	41	38
Orbitofrontal gyrus	45	0.001	5.61	27	53	−14
Middle frontal gyrus	23	0.001	5.60	−51	29	31
Superior frontal gyrus	148	0.003	5.38	−6	2	61
Inferior frontal gyrus	40	0.006	5.23	−45	11	28
**ARIPIPRAZOLE > HALOPERIDOL**
Middle temporal gyrus	10	<0.001	3.62	−66	−49	−5
Putamen	10	<0.001	3.44	−24	−16	−5
Middle frontal gyrus	5	0.001	3.28	36	47	−8

*The upper parts of the table show peak voxels of significant clusters from the within-group contrasts of Tower of London task > Control task (p_*FWE*_ < 0.05, *k* > 20). The lower part of the table shows results from an uncorrected exploratory analysis of the comparison aripiprazole > haloperidol (p_unc._ < 0.001, *k* > 5). The opposite contrast yielded no significant clusters. Coordinates are given in Montreal Neurological Institute and Hospital (MNI) coordinate system*.

According to our hypothesis, an ROI analysis was performed to test whether the activations in the frontal cortex, specifically targeted by the task, differed between the aripiprazole and the haloperidol groups. No significant differences were revealed with the ROI analysis. Exploratory uncorrected whole-brain analyses of aripiprazole > haloperidol and haloperidol > aripiprazole were performed at threshold level *p* < 0.001, *k* ≥ 5 (Table 3).

### Side Effects

Three subjects were unsuitable for scanning due to nausea, dizziness (aripiprazole), or claustrophobia (haloperidol). Three additional subjects reported nausea (aripiprazole). No subjects reported any side effects at one day or one week after participation in the study.

## Discussion

The present study shows a strong effect of the ToL task within the aripiprazole, haloperidol and placebo groups. The analyses revealed no differences in frontal cortical activation between the drug groups. Although aripiprazole and haloperidol have different pharmacodynamic profiles, the results may reflect similarities in their effect on brain activation. However, there might have been group differences that were too small to be detected in the present sample.

In the current study we used fMRI to directly compare aripiprazole- with haloperidol-affected frontal cortical activations. Few reports exist where neuroimaging has been used to investigate effects of aripiprazole in healthy individuals. One study showed a negative association between striatal D2 occupancy and frontal cortical metabolism after aripiprazole (Kim et al., 2013). A recent fMRI study employing the n-back task revealed decreased frontal activation after haloperidol, when compared to placebo and aripiprazole (Goozee et al., 2015). The current results cannot corroborate these findings as no significant differences in frontal cortical response were identified between the two drugs.

There are few previous studies investigating the effect of antipsychotics on cognition in healthy volunteers (Veselinovic et al., 2013). It has been shown that decreased performance in working memory is associated with increasing striatal D2 receptor occupancy (Kim et al., 2013). Although employing a different cognitive task, the current behavioral results are in line with this finding, indicating that increased haloperidol dose was associated with impaired task performance. Studies in patients and healthy volunteers have shown that typical antipsychotics are associated with larger cognitive impairments than atypical antipsychotics (Chung et al., 2012; Désaméricq et al., 2014), suggesting that haloperidol has a more detrimental effect on cognitive function than aripiprazole. However, some reports show a positive effect of haloperidol on cognitive performance (Mishara and Goldberg, 2004). In line with this, the present results show that the haloperidol group performed well and there was a tendency for better average performance than in the aripiprazole group. In the placebo group increased accuracy was associated with increased response times and decreased number of completed tasks. This relationship was not found in the drug groups, maybe indicating a less efficient performance. However, as the study was designed for fMRI, and not optimized for detecting behavioral differences, these findings are inconclusive.

The literature on dopaminergic manipulation in the frontal cortex in healthy individuals is scarce, and thus the current fMRI study of brain activation related to executive functioning is exploratory. Differences in brain response between the two drug groups were not identified, but it cannot be ruled out that the absence of group differences in this study may be due to lack of power. The drug effects on cognitive performance and related brain activity are dependent on baseline dopamine levels, and a between-subject design may not be optimal for identifying differential effects of the drugs. However, with repeated performance, the ToL task may be subject to training effects thus making a within-subject design suboptimal. The ToL task has been shown to yield different activation patterns that correspond to different task components (Newman et al., 2009). The components are not separated in the current version and it is possible that this task is not sensitive enough to reveal group differences. The advantage of the current ROI approach is that the ROI is specifically relevant to the task used; however it is possible that the approach may bias the group result if the placebo activation pattern cannot be generalized. Healthy subjects were used in the current study, and translating the findings to patients may be difficult as the present pharmacodynamic may differ from that found in illness. Also, the effect of an acute dose may differ from long-term treatment which is needed in patients, and different neurophysiological mechanisms may be involved (Grace et al., 1997). In contrast to studies conducted in patients, an advantage of the current study was that secondary effects of psychosis illness did not affect the results. However, potential consequences of side effects cannot be ruled out, and may differ between the two drugs. By using a crossover design future studies may increase the sensitivity, and a more standardized dosing protocol may also be beneficial. In addition, an event-related paradigm would make it possible to examine the activations in more detail.

Altered dopaminergic function in the striatum has been associated with reduced activity in the frontal cortex in schizophrenia patients and prodromal state individuals (Meyer-Lindenberg et al., 2002; Howes et al., 2009). The results of neuroimaging studies investigating the effects of antipsychotic drugs on activation in the frontal cortex are inconsistent, but most suggest that atypical antipsychotic drugs yield increased activation compared to typicals (Liemburg et al., 2012). The pharmacological profile of aripiprazole differs substantially from other atypical profiles, but very few neuroimaging studies have investigated aripiprazole. One fMRI study in schizophrenia patients employing an n-back working memory paradigm found improved activation in the frontal cortex after 4 weeks treatment with aripiprazole after switching from a typical antipsychotic (Schlagenhauf et al., 2010). In accordance with the Dopamine hypothesis aripiprazole is expected to show a net stimulating effect in the frontal cortex in patients with schizophrenia as its partial agonism property is thought to be present at low concentrations of endogenous dopamine (Howes and Kapur, 2009). Thus, one tentative explanation for the lack of difference in the present study may be that aripiprazole acts more antagonistic in the frontal cortex in healthy individuals than in schizophrenia patients, possibly resulting in activation differences that are too small to detect with the current design. Another interpretation relates to baseline dopamine levels. The PFC response to increasing cognitive load is thought to follow an inverted U-curve, and this is associated with dopamine transmission (Mattay et al., 2003). In patients with schizophrenia this curve is shifted leftwards (Egan et al., 2001). However, in healthy individuals, aripiprazole may induce a rightward shift in dopamine signaling leading to an impaired PFC response that is not differentiated from the effect of haloperidol. However, this is speculative, and more research is needed to elucidate the underlying mechanisms.

In conclusion, the current study revealed no significant differences in activation in the frontal cortex between aripiprazole challenge and haloperidol challenge in healthy volunteers performing the ToL task. This may be attributed to similarities between these two drugs’ effect on executive function in healthy individuals. However, the absence of group difference may also be caused by methodological limitations of the current study, and the issue needs further exploration.

## Author Contributions

JJ, IB and OAA conceived and designed the study, and BH designed the paradigm. IB and JJ were responsible for data acquisition, statistical analysis, execution of the study and wrote the first draft of the manuscript. IRG and AS participated in data acquisition and quality control. All authors are accountable for this work, contributed to revising the manuscript and have approved the final version.

## Conflict of Interest Statement

OA has received speaker’s honorarium from GlaxoSmithKline, Eli Lilly, Otsuka and Lundbeck. The other authors report no conflict of interest.

## Footnotes

^2^http://www.fil.ion.ucl.ac.uk/spm/
